# Synuclein Aggregate Assay Performance in Autopsy‐Confirmed Lewy Body Dementia: Results from the U.S. Dementia with Lewy Bodies Consortium

**DOI:** 10.1002/alz.088851

**Published:** 2025-01-09

**Authors:** James B Leverenz, Purvi Patel, Shane Formica, Lynn M. Bekris, Debby W Tsuang, Cyrus P Zabetian, Irene Litvan, Jori E Fleisher, Sarah Berman, David J Irwin, Andrea Bozoki, Carol Lippa, Oscar L. Lopez, Douglas R. Galasko, Dementia with Lewy Bodies Consortium

**Affiliations:** ^1^ Cleveland Clinic Lou Ruvo Center for Brain Health, Cleveland, OH USA; ^2^ Cleveland Clinic, Cleveland, OH USA; ^3^ Cleveland Clinic Genomic Medicine Institute, Cleveland, OH USA; ^4^ Cleveland Clinic Lerner Research Institute, Cleveland, OH USA; ^5^ Department of Psychiatry and Behavioral Sciences, University of Washington, Seattle, WA USA; ^6^ VA Puget Sound, Seattle, WA USA; ^7^ Geriatric Research, Education, and Clinical Center, VA Puget Sound Health Care System, Seattle, WA USA; ^8^ Geriatric Research, Education, and Clinical Center, Seattle, WA USA; ^9^ VAPSHCS/University of Washington, Seattle, WA USA; ^10^ University of California San Diego, La Jolla, CA USA; ^11^ Rush, Chicago, IL USA; ^12^ University of Pittsburgh, Pittsburgh, PA USA; ^13^ Department of Neurology, University of Pennsylvania, Philadelphia, PA USA; ^14^ University of North Carolina, Chapel Hill, NC USA; ^15^ Jefferson University, Philadelphia, PA USA; ^16^ VA San Diego Healthcare System, San Diego, CA USA; ^17^ University of California, San Diego, La Jolla, CA USA

## Abstract

**Background:**

Synuclein Aggregate Assays (SAA) in cerebrospinal fluid (CSF) and in skin biopsy have been shown to successfully identify underlying synuclein (Lewy body) pathology in patients with Parkinson's disease. Data in Lewy Body Dementia (LBD) is limited, particularly with pathologic confirmation and staging of underlying Lewy body pathology, and other co‐pathologies.

**Method:**

Utilizing data and biofluids from participants in the U.S. based Dementia with Lewy Bodies Consortium (DLBC) who have donated CSF to the study and come to autopsy, we examined the performance of CSF‐based SAA and AD biomarkers during life to post‐mortem presence and staging of Lewy body pathology (LBP) and Alzheimer's disease Neuropathologic Change (ADNC). SAA testing was performed on CSF by Amprion (SynTap).

**Result:**

Currently, the DLBC has enrolled over 160 participants, of which 31 cases have come to autopsy and been staged for LBP, ADNC, and TDP‐43 pathology. Twenty‐eight cases had CSF available from study participation. Three cases did not have LBP at autopsy and CSF SAA testing was negative in all three. Of the remaining 25 cases, two additional cases were CSF SAA indeterminate or negative and had anatomically limited LBP (brainstem and amygdala predominant respectively). One amygdala predominant LBP case had a positive CSF SAA. The remaining 22 limbic or neocortical stage LBP cases were CSF SAA positive ("detected"). SAA testing was positive in three cases with limbic or neocortical LBP and high stage ADNC co‐pathology.

**Conclusion:**

CSF‐based SAA testing performed well in Lewy body dementia with autopsy‐confirmed limbic or neocortical stage LBP. Detection of LBP when in more limited anatomic locations, brainstem or amygdala, was less reliable. False positive CSF SAA was not observed in this autopsy confirmed sample of LBD.

